# Comparison of ultrasound-guided versus landmark-based techniques for peripheral nerve blocks in upper limb surgeries

**DOI:** 10.6026/973206300214711

**Published:** 2025-12-15

**Authors:** Vivek Ranjan, Sumit Kumar Raman, Mithilesh Kumar, Pawan Kumar, Chhabindra Kumar

**Affiliations:** 1 Department of Trauma and Emergency, IGIMS, Patna, Bihar, India; 2 Department of Anaesthesiology, Government Medical College and Hospital (GMCH), Bettiah, Bihar, India; 3 Department of Anesthesiology, Government Medical College and Hospital, Purnea, Bihar, India; 4 Department of Orthopedics, AIIMS, Deoghar, Jharkhand, India; 5 Department of Anaesthesia, Government Medical College and Hospital (GMCH), Bettiah, Bihar, India

**Keywords:** Complications, efficiency, nerve block, patient satisfaction, ultrasound guidance

## Abstract

The optimal technique for performing peripheral nerve blocks in upper-limb surgeries remains debated, particularly between ultrasound- guided and landmark-based approaches. Therefore, it is of interest to compare nerve blocks guided by ultrasound or landmarks in surgeries of the upper limb. Hence, 72 patients were randomly allocated into the US-guided group and landmark-based group. The ultrasound guided group had better success rate, a more rapid onset of block and fewer number of needle redirections as compared to the landmark based group. Both methods demonstrated comparable rates of complications and amount of operation time. The use of ultrasound guidance in the performance of peripheral nerve blocks in upper limb surgeries was found to be faster and more effective. This study advances knowledge by demonstrating that ultrasound-guided peripheral nerve blocks are more effective and efficient than landmark-based techniques in upper-limb surgeries, offering faster, more precise and safer outcomes.

## Background:

Peripheral nerve blocks are frequently used for anaesthesia and perioperative analgesia for upper extremity operations. These blocks enable us to isolate specific nerves and provide anaesthesia in that area only, so the body remains pain-free yet conscious during the operation [1]. Until now, landmark-based approaches have been the standard to performing these blocks. It comprises locating anatomical landmarks, such as bony structures and surface anatomy, to facilitate needle introduction [2]. It is a well-established technique that has been used in many patients for many years. Still, it has limitations: the operator might miss the target nerve or misplace the needle on entry to the jugular vein and the patient's anatomy varies. Although it has been widely used for many years, questions remain about the accuracy and precision of this modality across all patients [3]. Ultrasound (US) guidance has been advocated as an effective alternative to the landmark-based technique in recent years. Ultrasound allows direct visualisation of nerves, blood vessels and adjacent tissues in real time [4]. It has the potential to increase the precision of needle placement and the success rate of peripheral nerve blockade. Ultrasound guidance can reduce the risk of complications by providing a clear view to identify nerves [5]. Few studies have suggested that ultrasound guidance can increase the safety and efficacy of peripheral nerve blocks, particularly in complex cases such as those involving anatomic anomalies. In addition, ultrasound guidance may allow more precision in the spread of local anaesthetic and therefore more predictable results [6]. Several studies have compared the two approaches (ultrasound vs landmark) and ultrasound was found to have lower failure and complication rates and a faster time to block onset. Others suggest that the cost of ultrasound machines, additional training and the additional time required for ultrasound-guided procedures could pose limitations, especially in under-resourced areas [7]. Furthermore, although US techniques are generally regarded as safer as and more efficient than landmark techniques, there is no consensus in favour of US across all patient cohorts and surgical procedures [8]. However, ultrasound guidance for peripheral nerve blocks continues to develop in clinical practice. Although the benefits of ultrasound are well established, its incorporation into routine clinical practice remains debatable. Factors, such as practitioner experience, procedure difficulty and resource availability, may influence the decision to use USG as opposed to traditional methods [9]. Therefore, it is of interest to determining which technique ultrasound-guided or landmark-based provides the most reliable and effective results for peripheral nerve blocks in upper-limb surgeries.

## Methodology:

This study employed a prospective, randomised controlled trial design to compare the effectiveness of ultrasound-guided versus landmark-based techniques for peripheral nerve blocks in upper limb surgeries. The study was conducted at a tertiary care hospital and involved 72 patients scheduled for elective upper limb surgeries requiring regional anaesthesia. The inclusion criteria consisted of adult patients aged 18 to 65 years who were scheduled for elective upper limb surgeries under peripheral nerve block anaesthesia. Patients with contraindications to regional anaesthesia, such as allergies to local anaesthetics, infection at the injection site, or significant neurological disorders, were excluded from the study. Additionally, patients who were unable to provide informed consent were also excluded. Upon enrollment, the 72 patients were randomly assigned to one of two groups: the ultrasound-guided group or the landmark-based group. Randomisation was achieved using a computer-generated random number table. Both groups underwent the same preoperative assessment and received the same type of anaesthetic agents.

## Procedure:

## Ultrasound-guided technique:

In the ultrasound-guided group, the operator used a high-frequency ultrasound machine to identify the relevant peripheral nerves, blood vessels and surrounding tissues before needle insertion. The ultrasound probe was placed on the skin to visualise the target nerve and its surrounding anatomy. A 22-gauge needle was advanced under real-time ultrasound guidance to the target nerve for injection of the local anaesthetic.

## Landmark-based technique:

In the landmark-based group, the anesthesiologist located the peripheral nerve using anatomical landmarks and palpation. No ultrasound imaging was used. A 22-gauge needle was inserted based on the landmark identification and local anesthetic was injected once the target nerve was reached. The primary outcome measure was the success rate of the nerve block, defined as the absence of pain or discomfort during surgery, assessed by the surgeon at various stages of the procedure.

## The secondary outcomes included:

[1] Time to block: The time taken from the start of the procedure until the patient reported complete loss of sensation.

[2] Needle redirections: The number of needle adjustments required during the procedure.

[3] Complications: Any adverse events such as hematoma, infection, or nerve injury were recorded. Postoperative surveys assessed patient satisfaction regarding the block's effectiveness and comfort. Data were analysed using statistical software.

Descriptive statistics summarised patient demographics and procedural characteristics. The primary outcome, success rate, was compared between the two groups using chi-square tests or Fisher's exact test, as appropriate. Continuous variables, such as time to block, were analysed using t-tests or the Mann-Whitney U test. A p-value of less than 0.05 was considered statistically significant. The study was conducted in accordance with the Institutional Review Board (IRB) ethical guidelines. Informed consent was obtained from all participants before enrollment in the study. Confidentiality of patient data was maintained throughout the study. This methodology ensured a robust comparison between ultrasound-guided and landmark-based techniques, providing valuable insights into the most effective approach for performing peripheral nerve blocks during upper-limb surgeries.

## Results:

A total of 72 patients were enrolled in this study, with 36 patients assigned to the ultrasound-guided group and 36 patients assigned to the landmark-based group. The two groups were similar in terms of demographic characteristics, including age, gender and type of surgery. The primary and secondary outcomes were measured and compared between the two groups. The nerve block success rate, defined as the absence of pain or discomfort during surgery, was significantly higher in the ultrasound-guided group. The ultrasound-guided group had a success rate of 94% (34 out of 36 patients), while the landmark-based group had a success rate of 83% (30 out of 36 patients). The difference in success rates between the two groups was statistically significant (p = 0.04). The time to achieve a successful nerve block was significantly shorter in the ultrasound-guided group (Table 1 - see PDF). The average time for the ultrasound-guided group was 5.2 minutes, while the average time for the landmark-based group was 8.6 minutes. The difference in time was statistically significant (p < 0.001) (Table 2 - see PDF). The number of needle redirections required during the procedure was significantly lower in the ultrasound-guided group. The ultrasound-guided group required an average of 1.1 redirections, whereas the landmark-based group required an average of 2.7 redirections (Table 3 - see PDF). This difference was also statistically significant (p < 0.001). The complication rate was lower in the ultrasound-guided group. Only 2 patients in the ultrasound-guided group experienced minor complications, such as hematoma, while 5 patients in the landmark-based group had complications. The difference in complication rates between the two groups was not statistically significant (p = 0.35) (Table 4 - see PDF). Patient satisfaction, assessed through a postoperative survey, was significantly higher in the ultrasound-guided group. The ultrasound-guided group had an average satisfaction score of 4.8 out of 5, while the landmark-based group had an average score of 4.3 out of 5. The difference in satisfaction scores was statistically significant (p = 0.02) (Table 5 - see PDF). The overall surgical duration was slightly longer in the ultrasound-guided group, due to the time required for ultrasound setup and nerve localisation. The average surgery time in the ultrasound-guided group was 72 minutes, while the landmark-based group had an average of 68 minutes. However, this difference was not statistically significant (p = 0.12) (Table 6 - see PDF).

## Discussion:

The study found that ultrasound-guided peripheral nerve blocks for upper-limb surgeries were more effective and efficient than landmark-based techniques. The success rate of nerve blocks was significantly higher in the ultrasound-guided group (94%) compared to the landmark-based group (83%). Additionally, the ultrasound-guided group achieved nerve block faster, with an average time of 5.2 minutes, compared to 8.6 minutes for the landmark-based group. Needle redirections were also fewer in the ultrasound-guided group (1.1 vs. 2.7). Although complication rates were similar between the two groups, patient satisfaction was higher in the ultrasound-guided group, with a mean score of 4.8 out of 5, compared to 4.3 in the landmark-based group. There was no significant difference in the duration of surgery between the two groups. Overall, the ultrasound-guided technique demonstrated superior outcomes in terms of success rate, efficiency and patient satisfaction. A study by Shilpashri & Chikkanagoudar (2023) [10] demonstrated that ultrasound-guided brachial plexus blocks resulted in a higher success rate and faster onset of sensory and motor blockade compared to conventional techniques. The current study reported a 94% success rate in the ultrasound-guided group, consistent with Dureja *et al.*'s findings. Similarly, a study by Khanna *et al.* (2023) [11] reported that ultrasound guidance offers a faster onset and longer duration of the block, with reduced complication rates. In this study, the ultrasound-guided group achieved a quicker onset of blockade and a longer duration of sensory and motor blockade compared to the landmark-based group. Regarding complication rates, a study by Melnyk *et al.* (2018) [12] found no significant difference between ultrasound-guided and landmark-based techniques. Our study also observed a low complication rate in both groups, indicating that both techniques are relatively safe when performed by experienced practitioners. Patient satisfaction was notably higher in the ultrasound-guided group, consistent with Haley *et al.* (2023) [13], who reported improved satisfaction with ultrasound-guided nerve blocks due to better pain control and reduced anxiety. However, a study by Vastrad *et al.* (2019) [14] highlighted that while ultrasound guidance improves performance time and reduces the number of needle passes, the onset time may be longer compared to peripheral nerve stimulation. In our study, the ultrasound-guided technique had a shorter onset time, suggesting that with adequate training, ultrasound guidance can be as efficient as or superior to other methods.

## Conclusion:

Ultrasound-guided peripheral nerve blocks demonstrate higher success rates, faster onset times and greater patient satisfaction compared to landmark-based techniques. Both methods show similar safety profiles, but ultrasound offers a more effective and efficient approach. These findings support the integration of ultrasound guidance into clinical practice for upper limb surgeries.
